# Dilute nitride and GaAs n-i-p-i solar cells

**DOI:** 10.1186/1556-276X-7-631

**Published:** 2012-11-20

**Authors:** Simone Mazzucato, Benjamin Royall, Richard Ketlhwaafetse, Naci Balkan, Joel Salmi, Janne Puustinen, Mircea Guina, Andy Smith, Russell Gwilliam

**Affiliations:** 1 School of Computer Science and Electronic Engineering, University of Essex, Colchester CO4 3SQ, UK; 2 Optoelectronic Research Centre, Tampere University of Technology, Tampere, 33720, Finland; 3 Surrey Ion Beam Centre, Advanced Technology Institute of Surrey, University of Surrey, Surrey, GU2 7XH, UK

**Keywords:** Dilute nitride, n-i-p-i solar cell, Ion implantation

## Abstract

We demonstrate for the first time the operation of GaInNAs and GaAs n-i-p-i doping solar cells with ion-implanted selective contacts. Multiple layers of alternate doping are grown by molecular beam epitaxy to form the n-i-p-i structure. After growth, vertical selective contacts are fabricated by Mg and Si ion implantation, followed by rapid thermal annealing treatment and fabrication into circular mesa cells. As means of characterisation, spectral response and illuminated current–voltage (*I*-*V*) were measured on the samples. The spectral response suggests that all horizontal layers are able to contribute to the photocurrent. Performance of the devices is discussed with interest in the n-i-p-i structure as a possible design for the GaInP/GaAs/GaInNAs tandem solar cell.

## Background

Currently, the highest efficiency solar cells are GaAs-based tandem solar cells (GaAs, GaInP and Ge) achieving efficiencies greater than 32% (AM 1.5 G at one sun spectrum) [1]. Multi-junction solar cells can achieve higher efficiency than single-junction cells as each junction is tuned to absorb a certain band of the solar spectrum. This reduces thermal relaxation and below-bandgap photon losses. In a triple-junction structure, the choice of bandgap of each material is critical in determining the maximum efficiency possible. The optimal bandgap for the bottom cell is 1 eV, but germanium with a bandgap of 0.67 eV is commonly used, limiting the efficiency of the tandem cell. Replacement of the Ge junction by dilute nitrides (Ga_0.91_In_0.09_N_0.03_As_0.97_ for example) with 1-eV bandgap could increase the theoretical limit for external efficiency from 42% to 52% at 1.5 AMD at 500 suns concentration [2] while remaining lattice-matched to GaAs.

For a solar cell to collect minority carriers effectively, the diffusion length of the bulk material should be comparable to the thickness of the active region of the device. However, the addition of nitrogen to GaAs greatly reduces the material quality with electron mobilities reported to be in the order of 300 cm^2^ V^−1^[3] and minority carrier lifetimes lower than 0.9 ns [4]. These values result in diffusion lengths of less than 0.8 μm, which is much shorter than the average thickness of 3 μm required for most GaAs-based solar cell devices to absorb nearly 90% of incident solar radiation in the desired range [5]. This poses a challenge in creating an efficient solar cell from GaInNAs using conventional designs. The short minority carrier lifetime and, hence, diffusion length are attributed to the incorporation of N in the GaInAs, which results in shallow recombination centres in the middle of the bandgap [6].

We attempt to address the problem of the short minority carrier lifetime in GaInNAs by the use of a n-i-p-i structure. Firstly, we tested the principle of this design on GaAs and later extended it to GaInNAs. However, GaAs does not have any diffusion length problem as the minority electron and hole diffusion lengths of 3 and 1.5 μm are comparable to the thickness required for efficient photon absorption.

**Figure 1:**
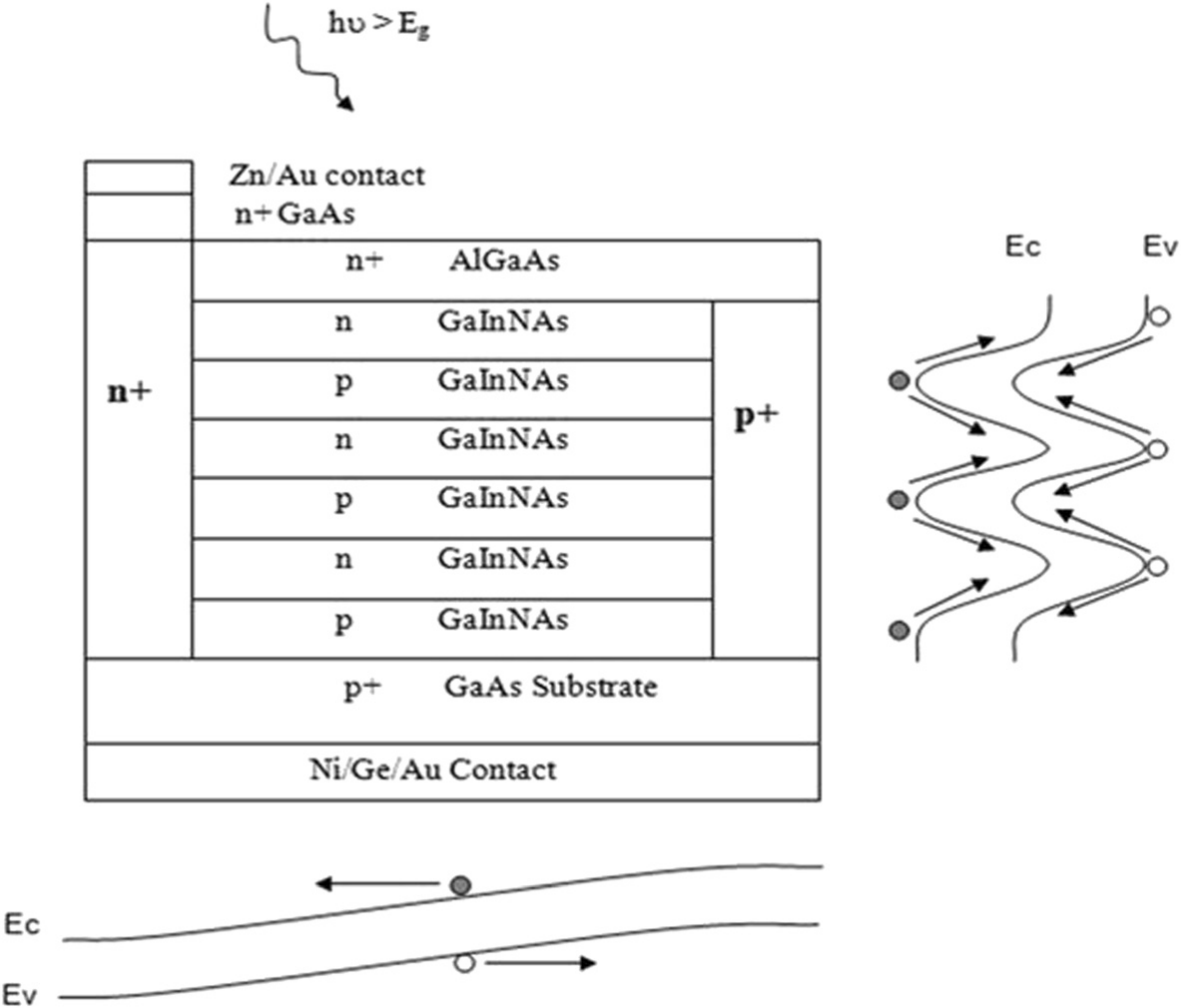
A prototype n-i-p-i device structure with highly doped lateral selective contacts for charge separation.

The n-i-p-i structure consists of a number of layers whose doping alternates between n-type, intrinsic, p-type and intrinsic [7-9]. This layer configuration gives rise to sinusoidal-like band alignment, which is represented in Figure 1. If the thickness of the layers is less than the diffusion length of the bulk material, photogenerated electron–hole pairs generated anywhere in the device will be rapidly separated, as a result of the minority carriers diffusing to an adjacent layer. The separated carriers then diffuse along the parallel layers towards the lateral selective contacts.

The n-i-p-i design is of particular interest for materials with short diffusion lengths, such as dilute nitrides. Since the horizontal layers' thickness is kept within the diffusion length of the bulk material, by repeating the doped layers, high absorption can be achieved without exceeding the diffusion length of the material. A significant challenge in creating a n-i-p-i solar cell is the creation of vertical selective contacts for electrons (holes), which will form ohmic contacts with n(p)-type layers and be rectifying to n(p)-type layers. Different techniques with their own merits and demerits have been demonstrated by different authors [8,9]. Here, n- and p-type selective contacts are created by ion implantation of Si and Mg, respectively.

In our previous work, we theoretically modelled dilute nitride n-i-p-i solar cells both alone and when incorporated as the third junction in a GaInP/GaAs/GaInNAs tandem solar cell [10]. Our models suggest that replacing the Ge layer in Ga_0.44_ In_0.56_P/GaAs/Ge with GaInNAs n-i-p-i subcell increases the efficiency of the tandem device to 35.7%, assuming 100% collection of majority carriers.

## Methods

### Device fabrication

The GaAs and GaInNAs n-i-p-i solar cells were grown by molecular beam epitaxy. The GaAs n-i-p-i solar cell had three junctions (a one-junction device would correspond to a conventional solar cell). The active region of the device has the following structure: 100-nm-thick n-type emitter, followed by a 300-nm p-type layer, a 600-nm p-type layer, a 1 μm-thick n-type layer and finally a 300-nm p-type base. The GaInNAs solar cell was a nine-junction n-i-p-i device with n- and p-type layers with thicknesses between 100 and 300 nm. These designs were derived from the drift diffusion model for n-i-p-i devices used in [10]. In both devices, a 40-nm Al_0.89_Ga_0.11_As window layer was grown on top of the n-type layer to reduce surface recombination. In addition, a 200-nm-thick highly doped n-type contacting layer was grown in all devices. The contacting layer was highly doped at 5 × 10^18^ cm^−3^ to ensure that it was not inadvertently compensated during the Mg ion implantation. In these two devices, the doping of the active layers was kept relatively low at 2 × 10^17^ cm^−3^ to allow them to be easily compensated during the creation of selective contacts.

**Figure 2:**
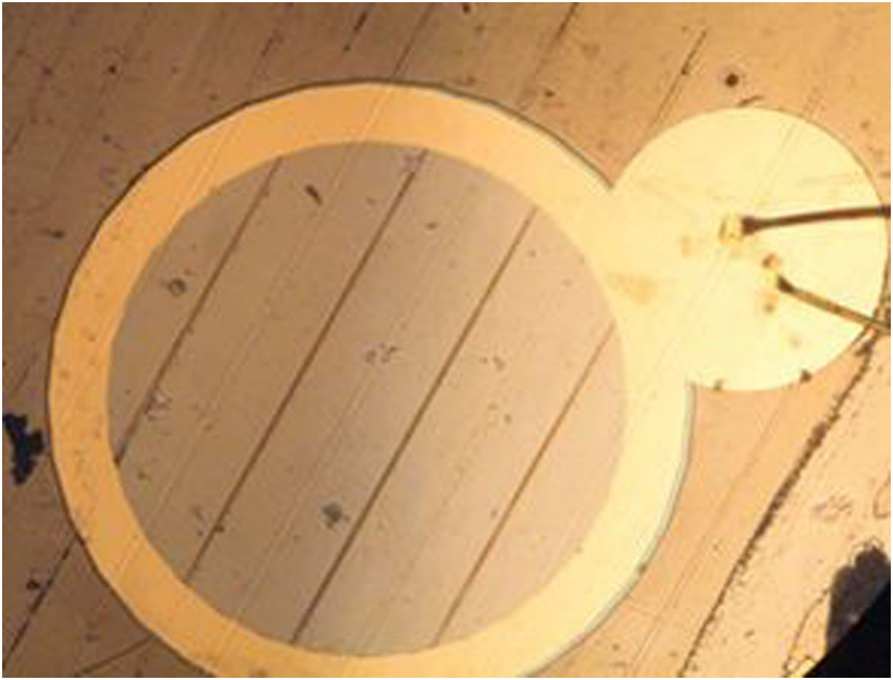
Photo of the fabricated n-i-p-i solar cell showing ion implant lines and standard top ring contacts.

After growth of the n-i-p-i devices, the implantation of Si and Mg ions was required to create the p- and n-type selective contacts, respectively. As selective contacts were required in different areas of the device, a nickel mask was applied to the devices which exposed the areas where Si implantation was required. After the Si implantation, the nickel mask was removed and then recreated to expose the areas where Mg implantation was required for the p-type contact. The spacing between the two ion implantations in both GaAs and GaInNAs devices was 100 μm. These appear as parallel lines on the sample surface (see picture of the fabricated cell in Figure 2) due to slight (10 nm) inadvertent etching of the GaAs contacting layer. The projected range of the ions was chosen so that the selective electrodes would extend between 240 nm and 2.2 μm in the device, doping all of the active layers. The samples were then treated via rapid thermal annealing for 10 s at 800°C for the GaInNAs cell and at 850°C for the GaAs one. After this, the devices were fabricated in a circular mesa structure, with a top 1-mm^2^-diameter optical aperture. AuGe/Ni/Au ring contact was evaporated onto the top of the device, while Zn/Au contact was deposited at the bottom. No antireflective coating was applied.

## Results and discussion

**Figure 3:**
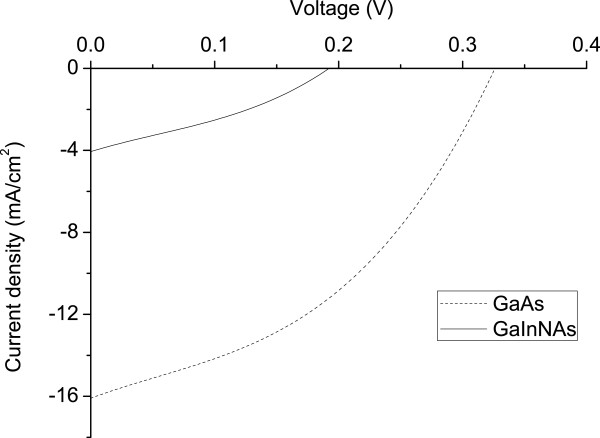
**The AM 1.5 G *J*-*V *characteristics for GaAs and GaInNAs n-i-p-i solar cells.**

The AM 1.5 G current density-voltage (*JV*) characteristic of the GaAs n-i-p-i solar cell is shown in Figure 3. The GaAs n-i-p-i cell recorded a short-circuit current density (*J*_sc_), open circuit voltage (*V*_oc_), fill factor (FF) and efficiency (*η*) of 16 mA/cm^2^ (accounting for grid coverage), 0.33 V, 54% and 2.2%, respectively. In our previous work, the best GaAs n-i-p-i device was annealed at 725°C and achieved *J*_sc_ = 12.9 mA/cm^2^, *V*_oc_ = 0.19 V and *η* = 0.87%, under the same illumination conditions [11]. Though we have not established optimum annealing conditions for these devices, the results suggest that the devices which annealed at a higher temperature of 800°C will show an improvement in all figures of merit.

The reflection losses have not been accounted for in calculating the current density; however, *J*_sc_ = 16 mA/cm^2^ is still much lower than the >29 mA/cm^2^ recorded with conventional GaAs solar cells [12].

Other recently demonstrated n-i-p-i devices show relatively high short-circuit currents and a disproportionately low *V*_oc_ and FF [8,9]. The cause of the low *V*_oc_ is not yet fully established. Results show that the *V*_oc_ varies to some extent with the fabrication technique and the quality of the selective contacts. Ion implantation of the selective contact offers the possibility of fabricating contacts on the front and back of the device without shorting it as the implants can start deep in the device. However, the introduction of dislocations at ion-implanted interfaces possibly increases recombination diffusion dark current, consequently reducing *V*_oc_. The re-grown contacts yield a slightly higher *V*_oc_[9]. However, with the re-grown contacts, the easiest technique is to have both contacts at the top of the cell, which can pose a problem in growing tandem structures.

**Figure 4:**
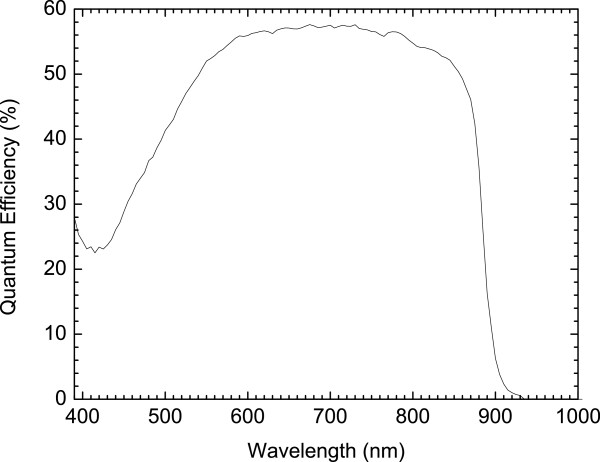
Spectral response of the GaAs n-i-p-i solar cell.

The spectral response of the GaAs n-i-p-i solar cell was obtained by illuminating the cell using a Bentham IL1 illuminator (Bentham Instruments Ltd., Reading, Berkshire, UK), followed by a Bentham M300 monochromator (Bentham Instruments Ltd.). The spectrum, shown in Figure 4, is typical of GaAs solar cells with the spectral response staying almost constant between the short wavelength cut-off due to the bandgap of the window layer and the long wavelength cut-off due to the bandgap of the cell. This suggests that minority carriers generated at all depths in the device contribute to the photocurrent. This can be inferred to all the layers of the device contributing to the photogenerated current.

**Figure 5:**
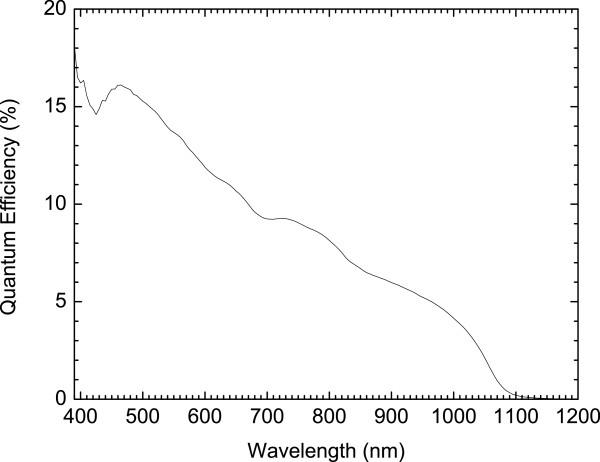
Spectral response of the GaInNAs n-i-p-i solar cell.

The spectral response profile of the GaInNAs n-i-p-i solar cell was taken using the same experimental conditions, and it is shown in Figure 5. As expected, it extends to longer wavelengths of 1.1 μm corresponding to photon energies of approximately 1.1 eV. The dropping shape of the spectrum suggests that only the top layers contribute to the device current. This could be purely a fabrication problem which has no bearing in the material properties or the design. If the ion implants have not reached or been activated at the bottom layers, the device will collect carriers at the top layers (short wavelengths) while most of the carries at the bottom layers (long wavelengths) will recombine before reaching the vertical contacts, consequently leading to low short-circuit current density values. As a result, the device will behave like a thin cell which only absorbs shorter wavelength photons, and it is transparent to the longer wavelength ones. In relation with the *J*-*V* curve, our GaInNAs device had *J*_sc_ = 4.2 mA/cm^2^ and *V*_oc_ = 0.19 V, as shown in Figure 3. The very low value of *J*_sc_ can be explained by the shape of the spectral response curve.

## Conclusion

The working of photovoltaic n-i-p-i structure has been demonstrated on GaAs and GaInNAs materials. The performance of the devices was measured with their *J*-*V* characteristics and spectral response profiles. The GaAs n-i-p-i solar cell devices have good carrier transport, and the best device generated a short-circuit current density of 16 mA/cm^2^ at room temperature; however, this still does not compare favourably with GaAs conventional-design solar cells at >29 mA/cm^2^. The performance of the devices can improve further with inclusion of antireflection coating and a back surface field. The square-like spectral response profile also suggests that minority carriers photogenerated anywhere in the devices contribute to the current. The dilute nitride (GaInNAs) n-i-p-i yielded a very low short-circuit current value of <5 mA/cm^2^. There was a possibility of some n-i-p-i layers not contributing to device current, hence yielding a very low value of the short-circuit current density. Both GaAs and GaInNAs n-i-p-i produced a low *V*_oc_ of 0.33 and 0.19 V, respectively, which is typical of currently demonstrated n-i-p-i devices. To consider inclusion of dilute nitride (GaInNAs), n-i-p-i as a subcell in tandem structures like the GaInP/GaAs/GaInNAs triple junction, the cause of disproportionately low *V*_oc_ in these devices has to be further investigated.

## Competing interests

The authors declare that they have no competing interests.

## Authors' contributions

JP and MG grew the sample, SM and JS fabricated the device and BR and AS performed the ion implantation. The experimental work was done by BR and RK under the supervision of SM and NB. Data analysis, calculation and manuscript conception was done by SM and BR. All authors read and approved the final manuscript.
